# A comparative analysis of routine techniques: Reverse transcriptase polymerase chain reaction (RT-PCR) and five cell lines for detection of enteroviruses in stool specimens

**Published:** 2011-06

**Authors:** F Abbasian, H Tabatabaie, M Sarijloo, S Shahmahmoodi, A Yousefi, T Saberbaghi, T Mokhtari Azad, R Nategh

**Affiliations:** Virology Division, Pathobiology Department, School of Public Health, Tehran University of Medical Sciences, Tehran, Iran.

**Keywords:** Enteroviruses, RT-PCR, Cell Cultures

## Abstract

**Background and objectives:**

Each year, Enteroviruses infect millions of people and cause different diseases. The agents are usually detected using cell culture. RD (Rhabdomyosarcoma) and L20B (L cells) are among the recommended cells by the World Health Organisation (WHO) for this purpose. Even though cell culture is the most common method used in diagnosing Enteroviruses in stool specimens, this particular method poses some problems, which include false positive or negative results, lack of a unique cell line for diagnosing all Enterovirus types in addition to being time consuming. For these reasons, an attempt was made to find better techniques of Enterovirus detection. RT-PCR (Reverse Transcriptase Polymerase Chain Reaction) is a technique used in place of the cell culture method. In this study, the cell culture method was compared with RT-PCR for detection of Enteroviruses in stool specimens.

**Material and method:**

First, the chloroform treated stool samples were inoculated onto five cell lines, including RD, L20B, Hep-2 (Human Epidermoid carcinoma cell line), Vero (Verde Reno) and GMK (Green Monkey Kidney). The results were then compared with data from Enterovirus detection using the RT-PCR technique.

**Results and conclusion:**

The difference between RT-PCR and cell culture results was significant. Enteroviruses were detected in 24% of specimens using RT-PCR while cell lines could isolate Enteroviruses in just 14.4% of the samples.

## INTRODUCTION

Based on the latest virus classification, human Enterovirus genus is divided into five species including Poliovirus (PV-1, -2 and -3), Human Enterovirus A (HEV-A) (Coxsackievirus A2, 3, 5, 7, 8, 10, 12, 14 and 16 and Enterovirus 71), HEV-B (Coxsackievirus A9, Coxsackievirus B 1–6, Echovirus 1–7, 9, 11–21, 24–27, 29– 33 and Enterovirus 69), HEV-C (Coxsackievirus A1-3, 11, 13, 15, 17–22 and 24) and HEV-D (Enterovirus 68 and 70) which can be transferred orally and infect the intestinal tract (1–3). Infections are usually not serious, but they can sometimes pass through intestinal cells and access inner parts of the body to cause some severe illnesses such as poliomyelitis, aseptic meningitis, myocarditis, foot and mouth disease, herpangina, pleurodynia, acute hemorrhagic conjunctivitis and other diseases (4–6).

After using animal cell cultures as a perfect technique for diagnosing Enteroviruses, it has become the gold standard for detection (4, 7). Attempts have been made to improve the techniques so as to find more sensitive cell cultures for virus detection and decrease cell infection rates. Nowadays, modern methods and materials such as air laminar flow systems, nanofilters and antibiotics reduce the rate of infection in the cells used (7). Meanwhile, calf bovine serum is used as cell supporter to ensure cell growth. Furthermore, inverted microscope has facilitated observations of cells and their cytopathic effects (CPE) (7, 8).

Cell culture technique, however, has some undeniable problems, and it needs to be improved. A special cell line can not support the growth of all viruses; on the other word, each virus can grow onsome special cell lines and a combination of cell lines are needed to isolate all serotypes of a big group of viruses such as Enteroviruses. For instance, although RD (Rhabdomyosarcoma) and L20B (L cell) are used for isolating Enteroviruses from stool specimens, they can not support all the serotypes. Furthermore, using a combination of at least two cell lines for isolation of Enteroviruses makes the technique time consuming and costly. In addition, cell culture contamination is a common problem that laboratories frequently encounter (9).

PCR is considered as an efficient method for virus detection at the moment (10, 11). The high speed involved in virus detection and its improved sensitivity makes the technique a favourite method for virus detection in cell cultures, clinical specimens, biopsy and autopsy (10, 11).

This study was aimed at comparing the cell culture method and RT-PCR (Reverse Transcriptase-PCR) for detection of Enteroviruses in the stool specimens. We have tried to increase the sensitivity of virus detection by using five cell lines simultaneously: RD, L20B, Hep-2 (Human Epidermoid carcinoma cell line), Vero (Verda Reno) and GMK (Green Monkey Kidney).

## MATERIALS AND METHODS

**Specimens**. 230 stool specimens, collected from patients with acute flaccid paralysis (AFP), were transferred to the laboratory under appropriate conditions.

**Preparation of the stool specimens**. Stool specimens were subjected to chloroform pre-treatment before inoculation to cell culture or genome extraction for RT-PCR. In addition to removing bacteria and fungi, chloroform pre-treatment removes potentially cytotoxic substance and dissociates virus aggregates (12).

**Cell Lines**. As the first step, all the cell lines used in this study (RD, L20B, Hep2, Vero and GMK) were evaluated for their sensitivity to Enteroviruses using known concentrations of vaccine Poliovirus, Echo11 and Coxsackievirus B and their sensitivity was confirmed. All the materials for cell culture, including cell culture media, fetal bovine serum and antibiotics, were prepared according to the standard procedure recommended by WHO (12). The treated samples were then inoculated onto monolayered cells prepared in cell culture tubes and were kept at 36°C (5% CO_2_and 80% humidity). The tubes were microscopically evaluated for 5 days to detect any evidence of cytopathic effect (CPE). To increase the sensitivity of virus isolation, blind passage was carried out on the cultures, which had remained negative, and they were checked for the next 5 days.

**Serotyping by Microneutralization**. In this study, microneutralization was performed based on the standard method recommended by WHO (World health organization) (12, 13).

**RNA extraction and RT-PCR**. RNA extraction and RT-PCR were performed as previously described (13, 14). Primers were prepared according to WHO protocol for identifying Enteroviruses (WHO, 2004) and can detect a conserved sequence in 5/ of the viral genome: EV-PCRI (5′–ACA CGG ACA CCC AAA GTA GTC GGT TCC –3′) and EV-PCR2 (5′–TCC GGC CCC TGA ATG CGG CTA ATCC-3′). The positive samples in cell cultures and neutralization test were used as positive controls.

First, a cycle was set for 20 minutes in 42°C for a reverse transcriptase reaction and 3 minutes in 95°C to deactivate this enzyme. Then, the programme contained 35 cycles (which included 45 seconds in 95°C, 45 seconds in 55°C, 45 seconds in 70°C and finally, 10 minutes in 70°C). The PCR product band (114 bp) was identified in Ethidium bromide containing agarose gel wells and by size marker with a molecular size number 8 (Roche) (12, 13).

**Statistical Analysis**. The data was analyzed by using SPSS, ANOVA test and Chi square tests. P-Value less than 0.05 was used to indicate statistical significance.

## RESULTS

In order to determine a more effective method for diagnosing Enteroviruses from faeces, 230 stool samples (suspected to AFP) were compared by using different routine methods: cell culture and RT-PCR. Totally, 33 out of 230 specimens (14.4%) were found to be positive for Enteroviruses by cell culture (Table 1).

**Table 1:** Result of cell culture for Enterovirus detection.

Cell line Isolated virus	Hep2	Vero	RD	L20b	GMK	Total
Echoviruses	11	9	12	0	7	12

Polioviruses	9	8	9	9	5	12

Coxsackieviruses	5	1	0	0	0	5

Unidentified Serotypes	4	0	4	0	1	4

Sum	29	16	25	9	13	33

Rate of Enterovirus Isolation	87.9%	48.5%	75.8%	27.3%	40%	100%

**AFP:** Acute Flaccid Paralysis; **RD:** Rhabdomyosarcoma cell Line; **L20B:** L20B cell line; **HEP-2:** Human Epidermoid cancer cell line; **vero:** Verda Reno cell line; **gMK:** Green Monkey Kidney cell line; **RT-PCR:** Reverse Transcriptase Polymerase Chain Reaction; **WHO:** World Health Organisation.

In this study, unanimous with other studies, no cell culture has been found to be able to support growth of all Enteroviruses (15, 16). Despite being the gold standard for detecting some viruses, the cell culture technique had some limitations and it sometimes failed to detect Enteroviruses due to inhibitors which exist in the specimens, especially when the specimen is faeces (17, 18). In the case of Enteroviruses, cell cultures need at least two weeks to detect the virus in the specimen and this is a good opportunity for the virus to contaminate the surrounding environment (19, 20).

**Fig. 1:**
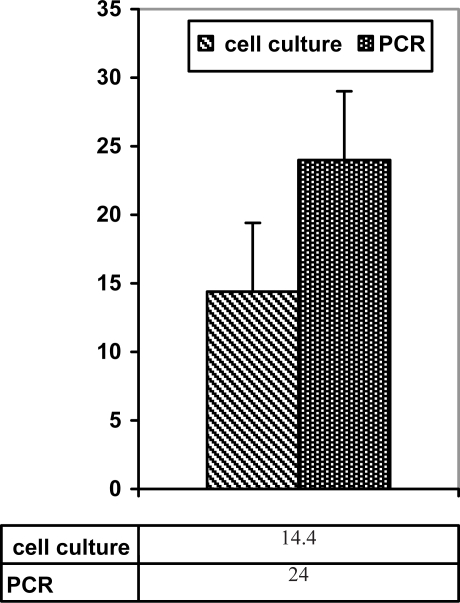
Comparing cell culture and RT-PCR methods for isolating Enteroviruses from stool samples.

For this reason, designing a rapid and highly sensitive method for diagnosing Enteroviruses in stool specimens was crucial to help the health system in detecting the agents much faster and more accurately. After setting up and using RT-PCR for detecting Enteroviruses in the specimens, the detection rate was improved to 24% (55 out of 230 specimens) (Fig. 1). Statistical analysis showed the differences in sensitivity as meaningful.

## DISCUSSION

Enteroviruses are one of the most important gastric viruses that can cause some dangerous diseases, especially in children (7). Although RD and Hep-2 are efficient cell lines for detection of most Enteroviruses, they can not support growth of all Enteroviruses (7, 15, 16). Furthermore, cell maintenance and some of the materials needed for cell culture (such as serum) are quite expensive, and checking cell cultures every day makes the technique boring and time consuming (19, 20).

To detect Enteroviruses in stool specimens by molecular methods, three kinds of pre-treatment of the specimen can be used: direct RNA extraction from stool specimen and then RT-PCR (1, 11, 21, 22), extraction of RNA from the stool specimens which have been pre-treated with chloroform (1, 11, 22–24), and RT-PCR on positive cell cultures of the stool specimens (23, 25, 26).In the present study, a single-step RT-PCR method for direct detection of Enteroviruses form stool samples was used as it is more cost effective and decreases the probability of cross contamination (10).

In the cell culture step, 5 cell lines (RD, L20B, Hep-2, GMK and Vero) were used to increase the probability of Enterovirus detection because several serotypes of Enteroviruses grow only in particular cell lines. By these cell lines, 33 cases out of 230 specimens (14.4%) were positive for Enteroviruses. Among all cell lines, RD and Hep-2 were able to detect more Enteroviruses. However, RT-PCR on pre-treated stool specimens could detect 55 Enteroviruses in 230 specimens (24%); much higher than Enterovirus detection rate in cell culture.

Other studies for comparing RT-PCR and cell culture in Enterovirus detection had same outcomes (4, 27, 28). They showed that different samples (4), the chosen procedure for RT-PCR, (27) the source of samples (27) and different conditions could affect the outcomes (4, 29). Although there are some differences in the results obtained, all studies have proven that RT-PCR is more sensitive than cell culture for Enterovirus detection. The findings in this study confirm the reports by others and have shown that RT-PCR makes the researcher more confident in detecting viruses in a variety of samples.

It is important to mention that PCR is a highly sensitive method and the procedure needs to be performed in a DNA free environment (30, 31). Utilizing separated areas for PCR ionic potential of solutions and concentrations of proper primers, nucleotides and polymerases are other factors that need to be taken into consideration when molecular methods are to be used for Enterovirus detection (30, 31). Obtaining false negatives in cell culture can be due to the presence of slow growing Enteroviruses in stool specimens, lack of sensitivity of the cell line, low titre of the virus in the specimens and toxic factors (17, 18, 30–32).
